# Diurnal variation in the performance of rapid response systems: the role of critical care services—a review article

**DOI:** 10.1186/s40560-016-0136-5

**Published:** 2016-02-24

**Authors:** Krishnaswamy Sundararajan, Arthas Flabouris, Campbell Thompson

**Affiliations:** Intensive Care Unit, Royal Adelaide Hospital and Discipline of Acute Care Medicine, University of Adelaide, Level 4, ICU, Robert Gerard Wing, Adelaide, 5000 South Australia Australia; Department of Medicine, University of Adelaide and the Royal Adelaide Hospital, Adelaide, 5000 South Australia Australia

**Keywords:** Intensive care unit, Afferent limb failure, Diurnal variation, Rapid response teams, Circadian variation

## Abstract

The type of medical review before an adverse event influences patient outcome. Delays in the up-transfer of patients requiring intensive care are associated with higher mortality rates. Timely detection and response to a deteriorating patient constitute an important function of the rapid response system (RRS). The activation of the RRS for at-risk patients constitutes the system’s afferent limb. Afferent limb failure (ALF), an important performance measure of rapid response systems, constitutes a failure to activate a rapid response team (RRT) despite criteria for calling an RRT.

There are diurnal variations in hospital staffing levels, the performance of rapid response systems and patient outcomes. Fewer ward-based nursing staff at night may contribute to ALF. The diurnal variability in RRS activity is greater in unmonitored units than it is in monitored units for events that should result in a call for an RRT. RRT events include a significant abnormality in either the pulse rate, blood pressure, conscious state or respiratory rate. There is also diurnal variation in RRT summoning rates, with most activations occurring during the day. The reasons for this variation are mostly speculative, but the failure of the afferent limb of RRT activation, particularly at night, may be a factor.

The term “circadian variation/rhythm” applies to physiological variations over a 24-h cycle. In contrast, diurnal variation applies more accurately to extrinsic systems. Circadian rhythm has been demonstrated in a multitude of bodily functions and disease states.

For example, there is an association between disrupted circadian rhythms and abnormal vital parameters such as anomalous blood pressure, irregular pulse rate, aberrant endothelial function, myocardial infarction, stroke, sleep-disordered breathing and its long-term consequences of hypertension, heart failure and cognitive impairment. Therefore, diurnal variation in patient outcomes may be extrinsic, and more easily modifiable, or related to the circadian variation inherent in human physiology. Importantly, diurnal variations in the implementation and performance of the RRS, as gauged by ALF, the RRT response to clinical deterioration and any variations in quality and quantity of patient monitoring have not been fully explored across a diverse group of hospitals.

**Electronic supplementary material:**

The online version of this article (doi:10.1186/s40560-016-0136-5) contains supplementary material, which is available to authorized users.

## Introduction

Timely patient assessment and effective triage, both have a major role in influencing the subsequent progress and outcome of acutely ill patients [1, 2]. Timely reviews by senior specialist physicians of new and acute patient admissions can be delayed [3]. There may also be inadequate oversight of a junior medical officer’s assessment and delivery of patient care, with the consequence of inefficiencies, inappropriate resource utilization and potential patient harm [4]. Senior clinicians may also fail to recognize acute deterioration and patterns of acute illness [3]. As a consequence, there can be a delay in formulating an appropriate plan, undertaking a procedure, instituting therapy or in imposing limits of care [5] for a potentially unstable inpatient.

Critical care areas provide critically ill patients with intense observation and treatment that cannot be provided on general wards [6]. These areas include intensive care units (ICUs), high-dependency units (HDUs), emergency departments (EDs) and operating theatres. Close monitoring enables early identification of patients with acute deterioration and the implementation of timely treatment by staff with critical care skills. In contrast, management of similar patients on general wards can be suboptimal and may be associated with higher mortality rates [3, 7].

The rapid response systems (RRSs) are becoming widely adopted. The RRS is the overarching system under which the rapid response team (RRT) operates. These teams evolved upon the basis that adverse events, such as deaths, cardiac arrests (CAs) and unanticipated ICU admissions, are often preceded by documented abnormalities in vital signs [8, 9] and that failure to respond to these signs is associated with increased mortality [10–12]. In the setting of an RRS, patients are identified when they meet one or more predefined criteria such as abnormalities in the heart rate, blood pressure, respiratory rate and neurological status.

The presence of any such criteria, or if a staff member is “worried” about the patient, is expected to trigger a prompt response from an RRT. Rapid response teams are staffed by clinicians with critical care skills who can assess and manage acute patient deterioration. The first described RRT, the medical emergency team (MET), was a critical care physician-led team [13]. Rapid response systems may therefore be physician led (MET) or nurse practitioner led (RRT and outreach teams) depending upon the hospital environment in which they operate. Since the advent of RRSs, cardiac arrests and associated mortality rates have fallen by up to 20–50 % in various institutions [14, 15] as well as across entire health regions [16].

Based on this premise, many safety and quality organizations have adopted the implementation of RRSs. In Australia, the Australian [17] Commission on Safety and Quality in Health Care (ACSQHC) has made the recognition of, and response to, deteriorating patients (standard 9) one of the 10 national standards (Additional file).

**Fig. 1:**
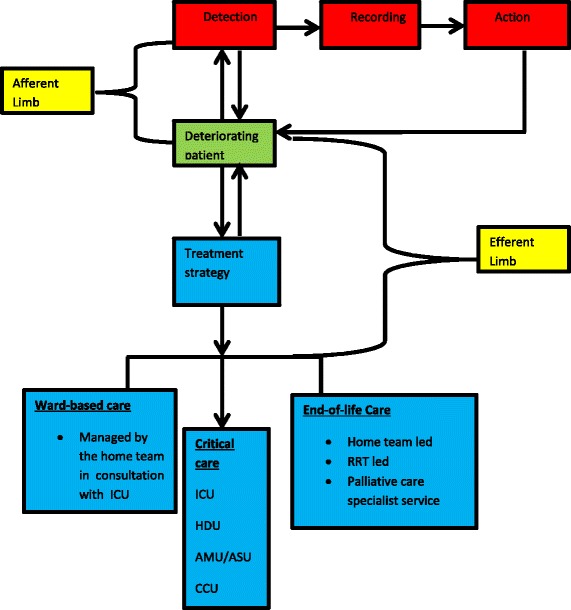
Diagram depicting the two limbs of the rapid response system

The RRSs have two key aspects: the afferent limb, which involves the detection, recognition of and response to acutely deteriorating patients, and the efferent limb, encompassing RRT patient assessment, management and dispatch (Fig. 1).

## Review

### Recognising the acute deteriorating patient

Medicine is becoming increasingly super-specialized, in part as a way of retaining expertise in the setting of ever expanding medical knowledge. Super-speciality medicine [18], by its nature, is restricted to a limited number of diagnoses, and has the benefits of better outcomes for those with specific conditions, particularly when super-speciality clinicians deliver care. However, patients and their clinical problems are becoming more diverse and complex [19], and those that die often have several co-morbid conditions.

Thus, patients are becoming less suitable for management by a super-specialized physician. In contrast, for the less complex and less well-differentiated patient, hospitalists (acute hospital medicine) can deliver a more efficient and complete service [20]. This does not mean that acute hospital medicine and super-speciality medicine are mutually exclusive. Some super-specialists are less likely to have the necessary skill set and infrastructure (i.e. monitoring environment) to provide acute medical care 24 h a day, 7 days a week, for patients who are critically unwell and are at risk of suffering an adverse event [20]. The RRSs were introduced to respond to acutely deteriorating patients [21] who in the past were “trapped” within the medical “silos” that have evolved with super-specialization.

Delay in the transfer of patients from the emergency department to intensive care is associated with a higher mortality [22]. Similarly, delays in the transfer of critically ill patients from the wards to the ICU and delays in responding to documented clinical deterioration are also associated with worse outcomes [23]. Patients recently discharged from an ICU are also at risk of a subsequent adverse event [24]. In this context, RRSs especially the critical care outreach teams behave as the “safety net” for the hospital at large.

Acute deterioration may be unexpected or go undetected. For example, the vast majority of in-hospital mortality can be accounted for by a small number of preceding conditions [25]. There are various scoring systems [21, 26] and tools [27–29] that utilize a combination of patient demographics, illness and biochemical measures to ascertain the risk of physiological deterioration or inpatient mortality. The deteriorating patient whose deterioration has not been recognized is at high risk of an adverse event (e.g. a cardiac arrest, unanticipated ICU admission, MET attendance) and associated morbidity.

It is also not uncommon for patients to have an adverse event despite having had a critical care review (e.g. MET or ICU) or despite having been discharged from a critical care area (e.g. ED, ICU or OR) in the preceding 24 h [30]. However, compared to an admitting team-only review, a critical care review is less likely to be associated with a subsequent adverse event [30].

## Responding to an acute deteriorating patient

**Fig. 2:**
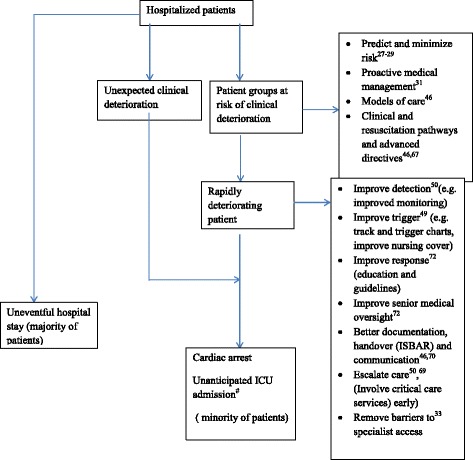
Flow diagram representing detecting and responding to clinical deterioration and afferent limb failure. (*#* indicates an admission to ICU which was not planned or elective; usually follows a sudden, unexpected clinical deterioration)

Adverse events are potentially preventable if patients’ vital signs are recorded in a timely fashion, are accurately documented, and there is an established RRS in place to respond to acute patient deterioration. Ward staff must recognize and respond promptly to abnormal patient vital signs, and trigger an RRT as appropriate. However, this process can, and does, fail at multiple levels. Even if abnormal observations are recorded and documented, their significance may not be recognized. Within that segmented structure, admitting teams, which are best at functioning within a narrow speciality paradigm, may fail to quantify accurately the risk of imminent death of their inpatients (Fig. 2).

Despite coming up with plausible diagnoses and treatment plans, medical teams may not call for help until the patient is moribund. Instead, inexperienced junior doctors are placed in a difficult position while liaising with interdisciplinary colleagues. In the quest for a unifying diagnosis, unnecessary investigations and consultations may distract clinicians from opportune treatment, including resuscitation.

## Failure to respond to an acute deteriorating patient: afferent limb failure

**Fig. 3:**
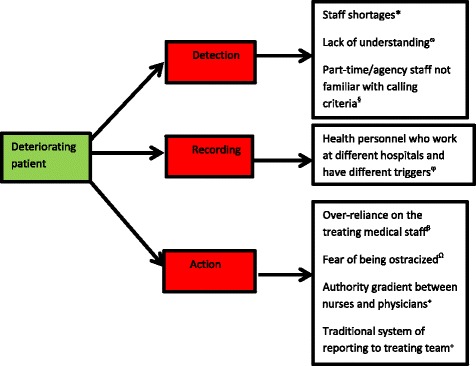
Contributors to afferent limb failure. * Davies et al. [76]. ø Tirkkonen et al. [77]. § Bragshaw et al. [78]. ϕ Galhotra et al. [79]. β Jacques et al. [80]. Ω Jones et al. [81]. α Azzopardi et al. [82]. ^+^ Radeschi et al. [83]

Even though the RRT system is well accepted in most hospitals, there are barriers to its full implementation. The hospital’s “cultural” awareness of an RRT and education of its healthcare personnel to demystify the concept of an RRT can positively impact upon the use of an RRT [31]. The expertise of the nursing staff, particularly its seniority and experience, may affect the rates of activation and rates of delayed/denied calling of the RRT [32]. Afferent limb failure (ALF) constitutes a failure to activate an RRT despite criteria for calling an RRT [33], and is an important performance measure of an RRS. Afferent limb failure can be an absolute phenomenon, wherein the RRT system is not activated at all. It could also be a relative concept, where the RRT system is activated, but activation is delayed relative to the actual or observed clinical deterioration (Fig. 3).

Afferent limb failure could occur at three stages: detection, recording and action. There may be a failure of detection of deranged vital signs [34]. For example, two Australian studies [35, 36] conducted after the implementation of the MET system identified afferent limb failure as a persistent problem.

In particular, the MERIT study [37], a large cluster randomized controlled study, showed that failure to detect a deteriorating patient and call an MET was common, despite documented MET criteria >15 min before the event, and occurred in 30 % of cardiac arrests, 51 % of unplanned ICU admissions and 50 % of unexpected deaths. Alternatively, there may be a failure to record patient vital signs. The respiratory rate is the most poorly recorded vital sign [38] and contributes to a significant proportion of ALF. Documentation of a complete set of vital signs is also often lacking. Only 17 % of surgical inpatients had a complete set of documentation of vital signs and a complete medical and nursing review within the first three post-operative days [39].

In addition to incomplete vital sign documentation, there may be a failure to document ward reviews by medical (14.9 %) and nursing (5.6 %) staff within the first seven post-operative days [40]. The final stage of afferent limb failure occurs at the level of MET criteria [39] where there is a failure to act on criteria and escalate [41] activation of the rapid response teams.

## Performance measurement of rapid response systems

Performance measurement of clinical systems is an important aspect of system maintenance, not only to ensure maximal efficiency and efficacy but also to improve patient outcomes [42]. The sustainability of any system whose aims include the prevention of adverse events is in part reliant upon a process of audit and feedback based upon agreed performance indicators [43].

For example, major trauma systems that evaluate the first responders to a critical event have swift feedback mechanisms in place that improve overall effectiveness by identifying areas of concern and then stimulating appropriate change [44].

Preferred measurements for evaluating the performance of RRS are still evolving. Commonly used measures are the rates of cardiac arrests and unanticipated admissions to the ICU from general wards [33]. In this context, ALF is a useful performance measure, as it is linked to a modifiable process.

## Dealing with afferent limb failure

Depending upon its cause, remedial measures are paramount in dealing with ALF (Fig. 2). For example, the detection of a deteriorating patient could improve with electronic monitoring of vital signs, particularly overnight [45]. A recent study [46] showed that the afferent limb of the rapid response system can be strengthened by an educational intervention (e-learning) specifically aimed at early detection of changes in vital signs. Having a tailored [46] management plan, not only for monitoring of vital signs but also for clinical handover, will help. This can be achieved, for example, by a structured clinical assessment and intervention focusing on the airway, breathing, circulation, disability and exposure or by reporting clinical deterioration using the ISBAR handover tool [46] (i.e. identity, situation, background, assessment and recommendation).

If staff shortages rather than staff performance are responsible for afferent limb failure, these can be remedied. Even if staff performance is responsible, it is also very important not to be critical of the ward staff who do not activate the MET appropriately or activate the MET inappropriately because this can affect team morale and productivity [47]. Process design rather than personal performance should be considered. A greater emphasis on repeated reviews of vulnerable patients is essential.

Even though it has been shown that ALF is associated with increased mortality [48], it remains to be fully elucidated as to how much of that mortality is due to issues surrounding delayed/absent decision-making in relation to end-of-life care. Sociologically informed models of interprofessional practice when dealing with cognitive and sociocultural aspects of ALF were shown to be helpful in dealing with ALF. The cognitive aspects contributing to ALF relate to perception (recording and measurement of vital signs), comprehension (how the vital signs relate to MET criteria and why) and projection (the clinical response required and the consequences). The sociocultural aspects revolve around the interpersonal and interprofessional aspects of the MET system.

Recently, there have been improved processes of care for recognizing the deteriorating patient with the help of education and widespread use of information tools [49, 50] such as posters, algorithms, electronic alerts. The most recent addition to this armamentarium is colour-coded track and trigger vital sign charts [49] that are based on the principle of patrolling surf lifesavers. It is imperative to evaluate these “between the flag” charts in terms of how they could influence the prevalence of ALF. Digital technology [50] has the potential to maximize the purported benefits from the track and trigger chart. What remains relatively unexplored is the effect of time of day upon the RRS performance and ALF in particular.

## Diurnal variation and the deteriorating patient

### Circadian variation and diurnal variation

The term “circadian variation” applies to physiological variations over a 24-h cycle. In contrast, diurnal variation as a concept applies more appropriately to extrinsic systems.

Circadian variation as defined by Franz Halberg [51] refers to daily rhythms that are endogenously regulated and repeated over a period of approximately 24 h in the absence of external stimuli. It is well known that the circadian system influences multiple human biochemical and physiological parameters, including sleep-wake cycles, thermoregulation, metabolic, endocrine and immune functions. Circadian rhythm has been demonstrated in an assortment of pathophysiological states. For example, there is an association between disrupted circadian rhythms and abnormal vital parameters (Table 1). There is also emerging evidence on the role of circadian misalignment and adverse consequences in patients admitted to an intensive care unit [52]. The environmental and genetic predisposition to maintenance and restoration of human circadian rhythms is a topic of ongoing research and still remains unexplored.

**Table 1:** Pathophysiological conditions that demonstrate diurnal variation

Anomalous blood pressure [84]	
Aortic dissection [84]	
Irregular pulse rate [85]	
Aberrant endothelial function [86]	
Increased platelet aggregation [86]	
Myocardial infarction [86]	
Stroke [86]	
Sleep-disordered breathing [87]	
Sympathetic overactivity [84]	
Impaired glucose tolerance [88]	
Adrenal insufficiency [89]	
Heart failure [86]	
Cognitive impairment [90]	

Diurnal variation, on the other hand, refers to the fluctuations that happen during the day and the variations in the day-night cycle that are not regulated by intrinsic or endogenous mechanisms but rather by extraneous factors. Thus, in the setting of the RRS performance, diurnal, rather than circadian, variation is more likely to be influenced by modifiable hospital processes.

## Diurnal variation in recognizing clinical deterioration

Staffing levels and expertise have an inverse relationship with patient outcomes [53]. There is consistent evidence to link diurnal variation with physician staffing and associated patient harm [54]. There is diurnal variation in the patient-physician ratio [55] and patient throughput (i.e. admission and discharge rates) in the ICU, this being maximal during day shifts and lower during night shifts.

In contrast, the ICU nurse-patient ratio may be more consistent throughout the day and night cycles. The mean nurse-patient ratio [55] was similar between day and night shifts with an average of 1.8 patients per nurse. On the contrary, physician-patient ratio [55] varied dramatically between day and night shifts, with a mean of 3.6 patients per physician during the day versus 8.5 patients per physician during the night. The impact of nurse-patient ratio in a general ward on ICU admissions has not been thoroughly evaluated across diverse hospitals and further research is needed.

There is also diurnal variation in patient outcomes. For example, outcomes for cardiac arrests, trauma [56], and elective and emergency surgery are worse at night. The relative role of extrinsic (diurnal) versus intrinsic (circadian) rhythms in these outcomes is unclear. Diurnal variation in shift times and duration also influences staff performance. Staff performance decreases during the night [57]. Also, patients admitted to an ICU during early morning hours tend to be older and sicker than those admitted later in the day [58]. The standard method of reporting RRT utilization rates is the number of RRT calls per 1000 patient admissions or discharges [13]. Afferent limb activation and rates of detection and response to clinical deterioration can, therefore, be expressed using the concept of MET dose [13]. Extending this analogy, we can describe a dose-response relationship, made obvious where there is diurnal variation in the MET dose. If we map cardiac arrest and RRT calls, their call pattern indicates a diurnal variation, whereby as the RRT dose decreases at night, the cardiac arrest rate increases [59]. It is important to ensure that this is not merely a chronological coincidence of a diurnal rhythm with a circadian one.

**Fig. 4:**
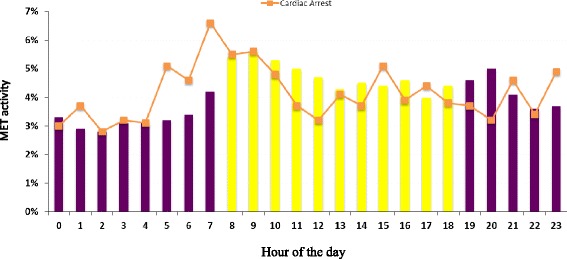
Diurnal variation in MET and cardiac arrest occurrence

**Fig. 5:**
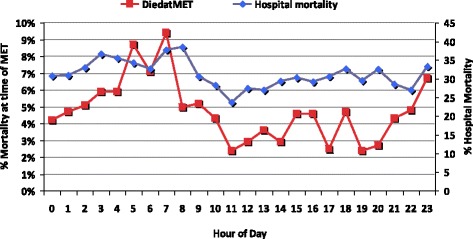
Diurnal variation in MET outcomes (based upon patients who had a MET call during their hospital stay)

There is a similar relationship between diurnal variation in the RRT dose and hospital mortality and outcomes at the time of an RRT call. Our experience in a tertiary referral centre mirrors previously published [59] data (Figs. 4 and 5).

In patients admitted to the ICU, there is an established link between overnight/weekend admissions and harm [60]. There is also evidence to suggest adverse outcomes among patients discharged after hours [61] from the ICU. A recent study [62] found that timing of discharge from ICU did not have an independent association with mortality, in contrary to previous studies. With regard to the RRS, further research is needed to explore and explain performance outcomes and their associations with diurnal variation.

## Diurnal variation in afferent limb failure

It may be that diurnal variation in the intensity with which inpatients are monitored or acute deterioration is responded to (e.g. ALF) may impact upon patient outcomes. There is a preponderance of RRT calls during the day. The reasons for this are mostly speculative, but the failure of the afferent limb of RRT activation, particularly at night, may be a factor. A large-scale retrospective observational study [63] demonstrated that the MET event rate was higher during the day than at night in unmonitored wards (62 % during the day vs. 38 % at night; *p* < .001) and monitored wards (59 % during the day vs. 41 % at night; *p* < .001) but not in the ICUs (47 % during the day vs. 53 % at night; *p* = .20). Unmonitored units had a greater daytime increase in MET event rate than monitored units (63 vs. 46 %), whereas the ICUs showed an 11 % decline in the MET event rate during the day compared with night. The day versus night difference was greater on weekdays (65 % during the day vs. 35 % at night; *p* < .001) than at weekends (56 % during the day vs. 44 % at night; *p* < .001) for MET activity in both monitored and unmonitored ward beds in the hospital.

A recent Australian [30] study identified that there were fewer RRT calls during the night than during the day (45 % of MET calls occur between 2000 and 0800 h). Even though ALF was prevalent, there was no diurnal variation in the pattern of ALF occurrence. Patients with afferent limb failure, compared to those without afferent limb failure, were significantly more likely to have an unanticipated ICU admission [36] (45/131 (34.4 %) versus 100/443 (22.5 %), *p* = 0.01). If there is a biological plausibility that major physiologic perturbations happen during the late night/early morning hours (Table 1) then, theoretically at least, there should be more MET calls during those hours. The absence of this pattern may either stem from afferent limb failure or the presence of another phenomenon that needs to be explored further.

## Diurnal variation in responding to clinical deterioration

Studies on diurnal variation in unanticipated ICU admissions, particularly regarding afferent limb failure and patient monitoring, are few. Patients admitted to hospitals after hours and at weekends have a higher observed and risk-adjusted mortality than patients admitted at other times [60, 64]. Current evidence is sparse with regard to the diurnal variation in the way we respond to acute deteriorations in patients who have to be cared for in hospital areas without the appropriate skill set. Delaying/deflecting admission to ICUs for this group of critically ill patients has been shown to be associated with worse outcomes [65].

From a health economics and risk management perspective, it is not unreasonable to have a 24/7 hospital-wide acute medical service [66] in addition to the critical care service. In particular, the response of a hospital’s acute services, e.g. trauma teams, critical care teams, RRTs, acute medical/acute surgical units and operation room (OR) availability with senior anaesthetist oversight, should be consistent across the day/night. In major hospitals during the day, a patient who has an RRT call gets the RRT team.

The RRT subsequently does a handover to the home team [67]. Overnight, the RRT operates [68] like a “hospitalist” service. That is, it sees any patient (no matter what the super-speciality home team is) and manages them in the absence of the home team. It may, if the complexity of the problem exceeds their and the ICU’s capacity or required super-speciality input, contact the home team overnight [62]. Otherwise, they deal with the issues and hand them over the next day to the home team. The burden of managing patients on the wards after hours in the absence of a member of the home team impacts significantly on the workload [55, 62] of the RRT and could divert them from their main role as “crisis managers”, which primarily revolves around troubleshooting clinical conundrums.

A hospitalist may work in parallel to the RRS in the early detection and response [69] to deteriorating patients, consistently across day and night time. Medically complex, elderly patients at risk of acute deterioration are more likely to populate acute hospitals. Increasing hospitalist workload has been associated with increased length of stay for patients and a high financial cost to the exchequer. In this scenario, the desire to maintain acute hospital [70] performance (e.g. shorter length of stay, greater patient throughput) will be accompanied by a greater demand for immediate access to critical care services.

## Challenges to hospital management at night: interface between RRT and hospitalists

The main challenge to hospital management at night would be the way the system deals with the sickest patients. These patients need the most astute doctors, and they need them at the right time. The hierarchical pyramid of a super-speciality consultant, doctors in training, interns, etc. may no longer provide efficient delivery of acute patient care. Clinicians must be comfortable dealing with diversity, complexity and chaos. The required skill set for this level of care is more often found among critical care and general medical/surgical physicians.

The transition [68] is already starting to occur. There are emerging data indicating that hospitalists [68] (i.e. generalists, general physicians), are more proficient in acute hospital care. Hospitals that employ hospitalists were potentially able to decrease the length of stay, minimize costs and improve mortality, without compromising patient outcomes or family satisfaction. Providing hospitalists [68] 24 h a day for 7 days a week is likely to be a major challenge for hospital management, particularly at night. The other important element is the environment in which acutely unwell inpatients are managed. Inpatients, regardless of their actual or perceived risk of deterioration, are often co-located (e.g. in a general ward). As a consequence, oversight of all types of patients may be equivalent, despite vast differences in their individual risks of an adverse event.

Thus, among RRSs, strategies have been developed to detect acute deterioration across the spectrum of inpatients (e.g. standardized patient observation and response charts). Despite the varying levels of evidence, the concept of locating undifferentiated/complex patients, within a critical care environment, coordinated and overseen by specialist physicians using a closed model is valid. Current evidence [69] reveals that inability to escalate care and thereby failure to rescue a deteriorating patient occurs in approximately 20 % of inpatients. Hospitalists [68] could potentially close the “treatment gap” and rescue such patients who could possibly fall between the cracks in the system.

## Challenges to hospital management at night: interface between RRT and palliative care

Recognizing medical futility and discussing the transition [70] from acute care to limited or palliative care based on accurate prognosis remains a challenge for both patients and clinicians, especially at night. There is a potential for therapy to become fragmented [71] and less tailored to the patient as a result of diurnal variation in the number and seniority of physicians available to make urgent clinical decisions.

Also, hospitals which have high nurse-staffing levels [71] achieve better satisfaction scores among patients, and this is an area for hospital administrators to be cognizant about, particularly with reference to the quality of clinical care. Improving senior medical oversight [72] at night with aims to improve system outcomes, ascertain medical futility, avoid inappropriate referrals, admissions to critical care and facilitate accurate prognostication is a way forward and the hospital at night [72] initiative is a positive step in that direction.

Patient and clinician expectations may not always be aligned [73], and this could pose difficulties in formulating a consensus on the medical management of a critically ill patient. The involvement of the rapid response teams in end-of-life decision-making [74] has also increased in recent times and, coupled with the diurnal variation in patients’ clinical condition and system issues [33] (i.e. afferent limb failure), management of patients in high-acuity ICU’s and hospitals, particularly at night [75], has become more complex and arduous.

## Implications

The overarching implications of diurnal variation within the RRSs and afferent limb failure, in particular, are that it impacts on the quality of care that patients receive. This literature review has shown that data are sparse on variations in outcomes through the 24-h day/night period. Variations, if they exist, might be physiological and unmodifiable. Equally, they may be diurnal and modifiable. However, we lack robust evidence to explain the complex interrelationship between circadian rhythm (intrinsic) and diurnal variation (extrinsic). Observational and interventional studies evaluating nocturnal surveillance and its association with resource limitations, circadian variation and confounding factors are needed.

## Conclusions

Diurnal variation exists in the activity of rapid response systems in the context of physiological circadian rhythms. Diurnal variation in the performance of hospitals, as measured by the quality and adequacy of patient monitoring, is a clear and immediate concern. Also, diurnal variation in the prevalence of afferent limb failure and its consequences has not been fully elucidated. The nexus between extrinsic hospital processes and innate human physiology across all critical and non-acute areas of a hospital in a 24-h period needs to be further investigated as this could potentially influence nocturnal patient management in hospitals.

## Additional file

Additional file 1:
**Australian Commission on Safety and Quality in Health Care (ACSQHC) standards.** (PDF 1013 kb)

## Abbreviations

AMU: Acute Medical Unit
ASU: Acute Surgical Unit
CCU: Coronary Care Unit
OR: Operating Room
HDU: High Dependency Unit
RRT: rapid response teams
ALF: afferent limb failure
ICU: intensive care unit
MET: medical emergency team
RRSs: rapid response systems
