# Supplementary material for: Diurnal variation in the performance of rapid response systems: the role of critical care services—a review article
Source: J Intensive Care. 2016 Feb 24;4:15. doi: 10.1186/s40560-016-0136-5 (PMC4765019; doi:10.1186/s40560-016-0136-5)
Supplement: Additional file 1: — Australian Commission on Safety and Quality in Health Care (ACSQHC) standards. (PDF 1013 kb) [file 40560_2016_136_MOESM1_ESM.pdf]

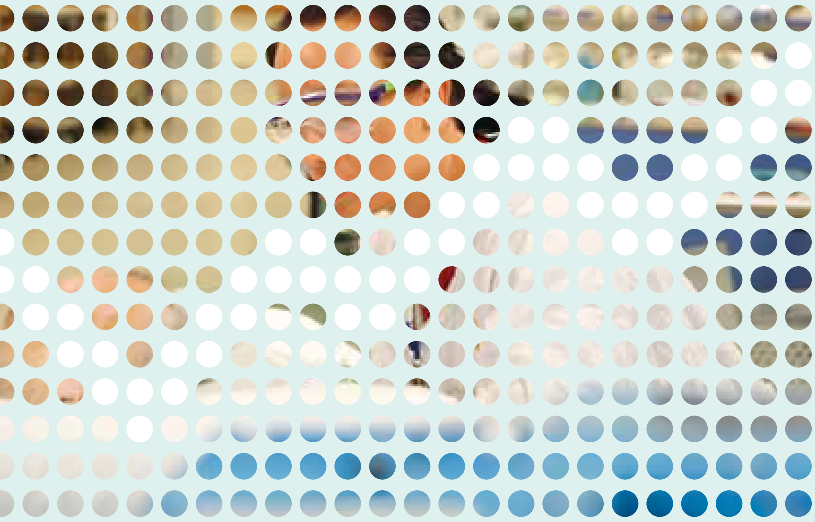

national consensus statement:

essential elements for recognising & responding to clinical deterioration

AUSTRALIAN COMMISSION ON SAFETY AND QUALITY IN HEALTH CARE

recognising + responding TO CLINICAL DETERIORATION

22 april 2010

ISBN: 978-0-9806298-8-0

© Commonwealth of Australia 2010

This work is copyright. It may be reproduced in whole or part for study or training purposes subject to the inclusion of an acknowledgment of the resource. Reproduction for purposes other than those indicated above requires the written permission of the Australian Commission on Safety and Quality in Health Care (ACSQHC).

ACSQHC was established in January 2006 by the Australian Health Ministers to lead and coordinate improvements in safety and quality in Australian health care.

Copies of this document and other work of ACSQHC can be found at www.safetyandquality.gov.au. For further information about the National Consensus Statement or the work of ACSQHC, contact the Office of the Australian Commission on Safety and Quality in Health Care on telephone: **+61 2 9263 3633** or email to: mail@safetyandquality.gov.au

Recommended Citation

Australian Commission on Safety and Quality in Health Care (2010). National Consensus Statement: Essential Elements for Recognising and Responding to Clinical Deterioration. Sydney, ACSQHC.

Acknowledgments

ACSQHC acknowledges the significant contribution of the Recognising and Responding to Clinical Deterioration Advisory Committee for their time and expertise in the development of this Statement.

ACSQHC also acknowledges the contributions of the many health professionals and organisations that provided comment and other support to the development of this Statement.

ACSQHC gratefully acknowledges the kind permission of St Vincent's and Mater Health Sydney to reproduce the images in this document.

national consensus statement:

essential elements for recognising & responding to clinical deterioration

22 april 2010

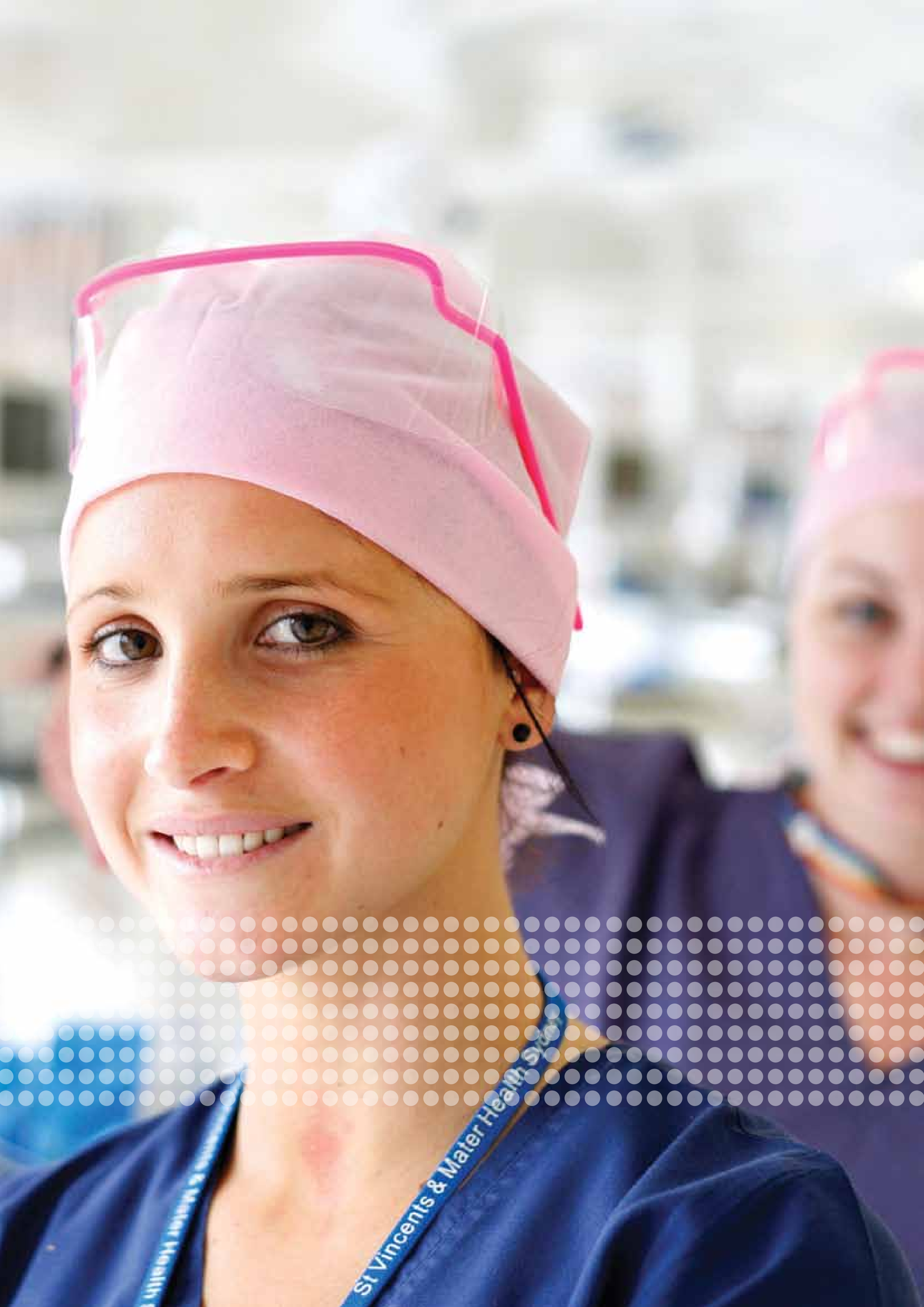
St Vincents & Mater Health Sydney

contents

Introduction 4

Scope 5

Intended audience 5

Guiding principles 6

Essential elements 7

A. Clinical Processes 8

1. Measurement and documentation of observations 8
2. Escalation of care 9
3. Rapid response systems 10
4. Clinical communication 12

B. Organisational Prerequisites 13

5. Organisational supports 13
6. Education 14
7. Evaluation, audit and feedback 15
8. Technological systems and solutions 16

Glossary 19

Contributing Documents 20

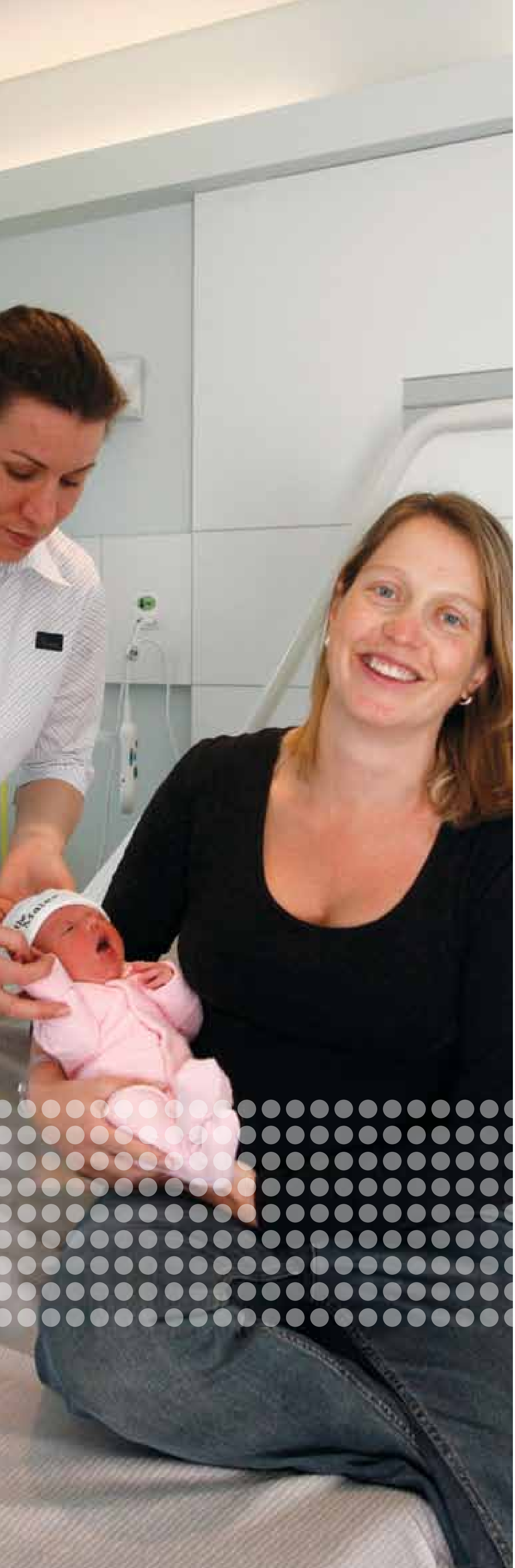

introduction

Early recognition of clinical deterioration, followed by prompt and effective action, can minimise the occurrence of adverse events such as cardiac arrest, and may mean that a lower level of intervention is required to stabilise a patient.

The evidence base regarding recognition and response systems for clinical deterioration is still developing. This document has been developed as a consensus statement reflecting the agreed views of experts in the field and the Australian Commission on Safety and Quality in Health Care. It has been derived from expert experience and published evidence. Guidelines and documents that have informed the Consensus Statement are listed at the end of the Statement on page 20.

In April 2010, Health Ministers endorsed the Consensus Statement as the national approach for recognising and responding to clinical deterioration in acute care facilities in Australia.

The purpose of the Consensus Statement is to describe the elements that are essential for prompt and reliable recognition of, and response to, clinical deterioration in acute health care facilities in Australia.

The Statement sets out agreed practice for recognising and responding to clinical deterioration. To achieve this, facilities need to have systems in place to address all elements in the Statement. As a consensus statement, the document represents guidance, rather than mandatory practice.

The Consensus Statement should guide health services in developing their own recognition and response systems in a way that is tailored to their patient population and the resources and personnel available, whilst being in line with relevant jurisdictional or other programs.

A glossary of terms used in this Statement appears on page 19.

scope

The Consensus Statement relates to situations where a patient's physiological condition is deteriorating. The general provision of care in a hospital or other facility is outside the scope of this document.

The Consensus Statement focuses on ensuring that a clinical safety net is in place for patients whose condition is deteriorating, and outlines the organisational supports that are needed to provide this safety net. It does not cover the specific clinical treatments or interventions that may be needed to stabilise a patient.

The Consensus Statement applies to all patients in an acute health care facility, including adults, adolescents, children and babies.

Within the context of the focus on physiological deterioration, the Consensus Statement applies to all types of patients, including medical, surgical, maternity and mental health patients.

The Consensus Statement applies in all types of acute health care facilities, from large tertiary referral centres, to small district and community hospitals. Some elements of the Consensus Statement may be used by services delivered by acute health care facilities in the community (such as hospital in the home programs).

intended audience

The Consensus Statement has been developed for:

- clinicians and managers responsible for the development, implementation and review of recognition and response systems in individual facilities or groups of facilities.
- planners, program managers and policy makers responsible for the development of jurisdictional or other strategic programs dealing with recognition and response to clinical deterioration.

guiding principles

1. Recognising patients whose condition is deteriorating and responding to their needs in an appropriate and timely way are essential components of safe and high quality care.

2. Recognition and response systems must apply to all patients, in all patient care areas, at all times.

3. Primary responsibility for caring for the patient rests with the attending medical officer or team. Recognition and response systems should therefore promote effective action by ward staff and the attending medical officer or team. This includes calling for emergency assistance when required.

4. Effectively recognising and responding to deterioration requires appropriate communication of diagnosis, including documentation of diagnosis in the health care record.

5. Effectively recognising and responding to deterioration requires development and communication of plans for monitoring of observations and ongoing management of the patient.

6. Recognition of and response to deterioration requires access to appropriately qualified, skilled and experienced staff.

7. Recognition and response systems should encourage a positive, supportive response to escalation of care, irrespective of circumstances or outcome.

8. Care should be patient focused and appropriate to the needs and wishes of the individual and their family or carer.

9. Organisations should regularly review the effectiveness of the recognition and response systems they have in place.

essential elements

These elements describe the essential features of the systems of care for recognising and responding to clinical deterioration. The elements do not prescribe how this care should be delivered. Facilities need to have systems in place to address all elements in the Consensus Statement; however the application of the elements in an individual facility will need to be carried out in a way that is relevant to its specific circumstances.

This Statement includes eight essential elements. Four relate to clinical processes that need to be locally delivered, and are based on the circumstances of the facility in which care is provided. Four relate to the structural and organisational prerequisites that are essential for recognition and response systems to operate effectively.

a Clinical processes

1. Measurement and documentation of observations
2. Escalation of care
3. Rapid response systems
4. Clinical communication

b Organisational prerequisites

5. Organisational supports
6. Education
7. Evaluation, audit and feedback
8. Technological systems and solutions

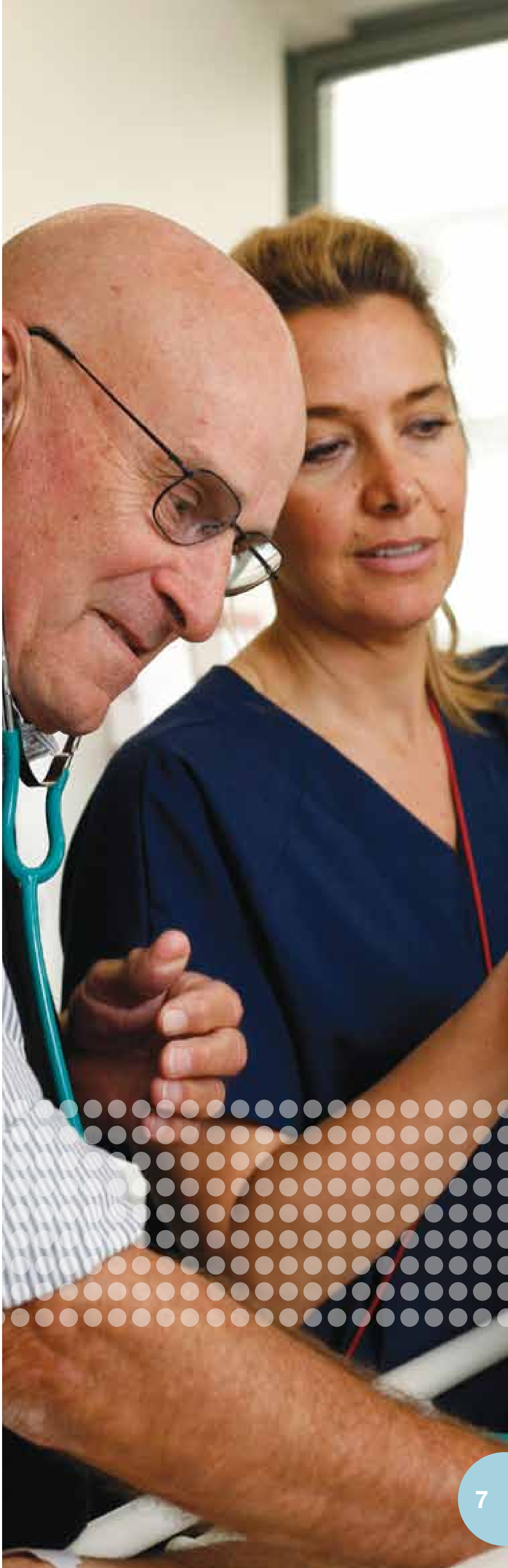

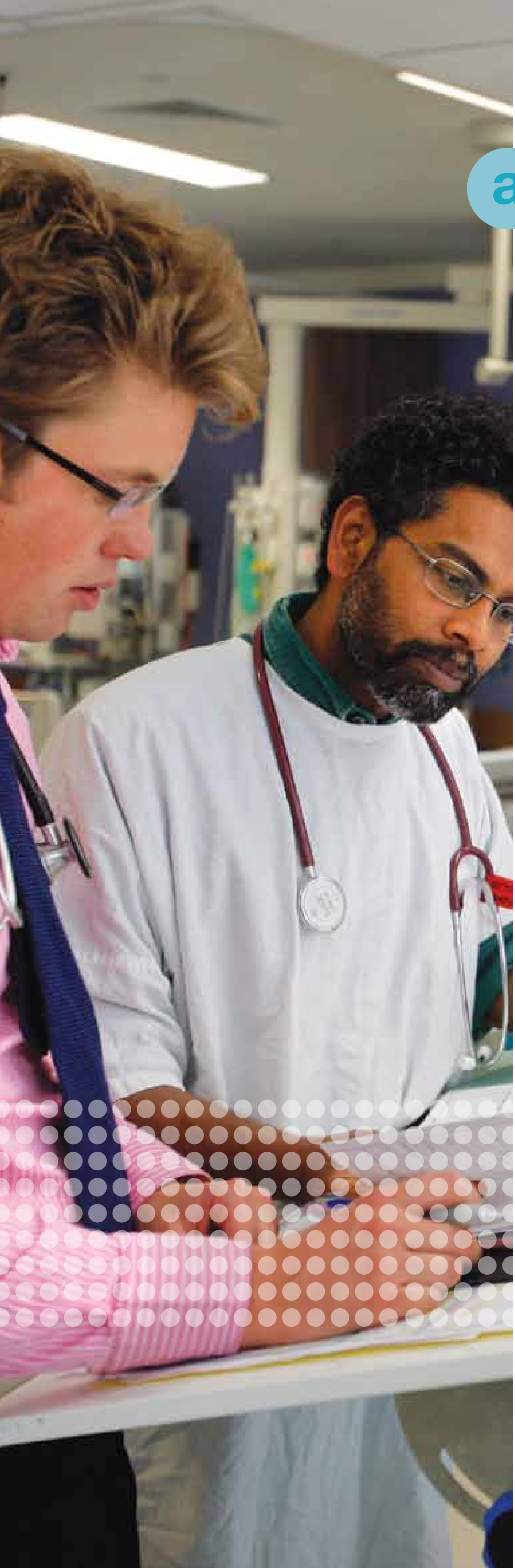

a clinical processes

1 Measurement & documentation of observations

Measurable physiological abnormalities occur prior to adverse events such as cardiac arrest, unanticipated admission to intensive care and unexpected death. These signs can occur both early and late in the deterioration process. Regular measurement and documentation of physiological observations is an essential requirement for recognising clinical deterioration.

1.1 Observations should be taken on all patients in acute care settings.

1.2 Observations should be taken on patients at the time of admission or initial assessment.

1.3 For every patient, a clear monitoring plan should then be developed that specifies the physiological observations to be recorded and the frequency of observations, taking into account the patient's diagnosis and proposed treatment.

1.4 The frequency of observations should be consistent with the clinical situation of the patient. For the majority of patients in an acute health care facility, observations should be taken at least once per eight hour shift. In some clinical circumstances more frequent or less frequent observations will be appropriate and this should be documented in the monitoring plan.

1.5 The frequency of observations should be reconsidered and possibly modified according to changes in clinical circumstances.

1.6 Physiological observations should include:

- respiratory rate
- oxygen saturation
- heart rate
- blood pressure
- temperature
- level of consciousness

In some circumstances, and for some groups of patients, some observations will need to be

measured more or less frequently than others, and this should be specified in the monitoring plan.

1.7 The minimum physiological observations should be documented in a structured tool such as an observation chart.

1.8 Observation charts should display information in the form of a graph. An observation chart should include:

- a system for tracking changes in physiological parameters over time
- thresholds for each physiological parameter or combination of parameters that indicate abnormality
- information about the response or action required when thresholds for abnormality are reached or deterioration identified
- the potential to document the normal physiological range for the patient

1.9 Clinicians may choose to document other observations and assessments to support timely recognition of deterioration. Examples of additional information that may be required include fluid balance, occurrence of seizures, pain, chest pain, respiratory distress, pallor, capillary refills, pupil size and reactivity, sweating, nausea and vomiting, as well as additional biochemical and haematological analyses.

2 Escalation of care

An escalation protocol sets out the organisational response required in dealing with different levels of abnormal physiological measurements and observations. This response may include appropriate modifications to nursing care, increased monitoring, review by the attending medical officer or team or calling for emergency assistance from intensive care or other specialist teams.

Primary responsibility for caring for the patient rests with the attending medical officer or team. In this context, the escalation protocol describes the additional safety net that must exist for all patients. Although this safety net should be tailored to the circumstances of the facility, it should include some form of emergency assistance where advanced life support can be provided to patients in a timely way. A protocol regarding escalation of care is an essential requirement for responding appropriately to clinical deterioration.

2.1 A formal documented escalation protocol is required that applies to the care of all patients at all times.

2.2 The escalation protocol should authorise and support the clinician at the bedside to escalate care until the clinician is satisfied that an effective response has been made.

2.3 The escalation protocol should be tailored to the characteristics of the acute health care facility, including consideration of issues such as:

- size and role (such as whether a tertiary referral centre or small community hospital)
- location
- available resources (such as staffing mix and skills, equipment, remote telemedicine systems, external resources such as ambulances)
- potential need for transfer to another facility

2.4 The escalation protocol should allow for a graded response commensurate with the level of abnormal physiological measurements, changes in physiological measurements or other identified deterioration. The graded response should incorporate options such as:

- increasing the frequency of observations
- appropriate interventions from the nursing and medical staff on the ward

- review by the attending medical officer or team
- obtaining emergency assistance or advice
- transferring the patient to a higher level of care locally, or to another facility

2.5 The escalation protocol should specify:

- the levels of physiological abnormality or abnormal observations at which patient care is escalated
- the response that is required for a particular level of physiological or observed abnormality
- how the care of the patient is escalated
- the personnel that the care of the patient is escalated to, noting the responsibility of the attending medical officer or team
- who else is to be contacted when care of the patient is escalated
- the timeframe in which a requested response should be provided
- alternative or back up options for obtaining a response

2.6 The way in which the escalation protocol is applied should take into account the clinical circumstances of the patient, including both the absolute change in physiological measurements and abnormal observations, as well as the rate of change over time for an individual patient.

2.7 The escalation protocol may specify different actions depending on the time of day or day of the week, or for other circumstances.

2.8 The escalation protocol should allow for the capacity to escalate care based only on the concern of the clinician at the bedside in the absence of other documented abnormal physiological measurements ('staff member worried' criterion).

2.9 The escalation protocol should allow for the concerns of the patient, family or carer to trigger an escalation of care.

2.10 The escalation protocol should include consideration of the needs and wishes of patients with an advance care directive or where other treatment-limiting decisions have been made.

2.11 The escalation protocol should be promulgated widely and included in education programs.

3 Rapid response systems

Where severe deterioration occurs it is important to ensure that the capacity exists to obtain appropriate emergency assistance or advice prior to the occurrence of an adverse event such as a cardiac arrest. Different models that have been used to provide this assistance include medical emergency teams (METs), critical care outreach and intensive care liaison nurses. The generic name for this type of emergency assistance is a 'rapid response system'. The emergency assistance provided as part of a rapid response system is additional to the care provided by the attending medical officer or team.

For most facilities, the rapid response system will include clinicians or teams located within the hospital who provide emergency assistance (such as a MET or a nurse accredited in advanced life support). In some facilities the system may be a combination of on-site and external clinicians or resources (such as the ambulance service or local general practitioner). However comprised, and however named, a rapid response system should form part of an organisation's escalation protocol.

3.1 Some form of rapid response system should exist to ensure that specialised and timely care is available to patients whose condition is deteriorating.

3.2 Criteria for triggering the rapid response system should be included in the escalation protocol.

3.3 The nature of the rapid response system needs to be appropriate to the size, role, resources and staffing mix of the acute health care facility.

3.4 The clinicians providing emergency assistance as part of the rapid response system should:

- be available to respond within agreed timeframes
- be able to assess the patient and provide a provisional diagnosis
- be able to undertake appropriate initial therapeutic intervention
- be able to stabilise and maintain the patient pending definitive disposition
- have authority to make transfer decisions and to access other care providers to deliver definitive care

3.5 As part of the rapid response system there should be access, at all times, to at least one clinician, either on-site or in close proximity, who can practise advanced life support.

3.6 The clinicians providing emergency assistance should have access to a staff member of sufficient seniority to make treatment-limiting decisions. Where possible these decisions should be made with input from the patient, family and the attending medical officer or team.

3.7 In cases where patients need to be transferred to another site to receive emergency assistance, appropriate care needs to be provided to support them until such assistance is available.

3.8 When a call is made for emergency assistance, the attending medical officer or team should be notified as soon as practicable that the call has been made, and where possible they should attend to support and learn from the clinicians providing assistance.

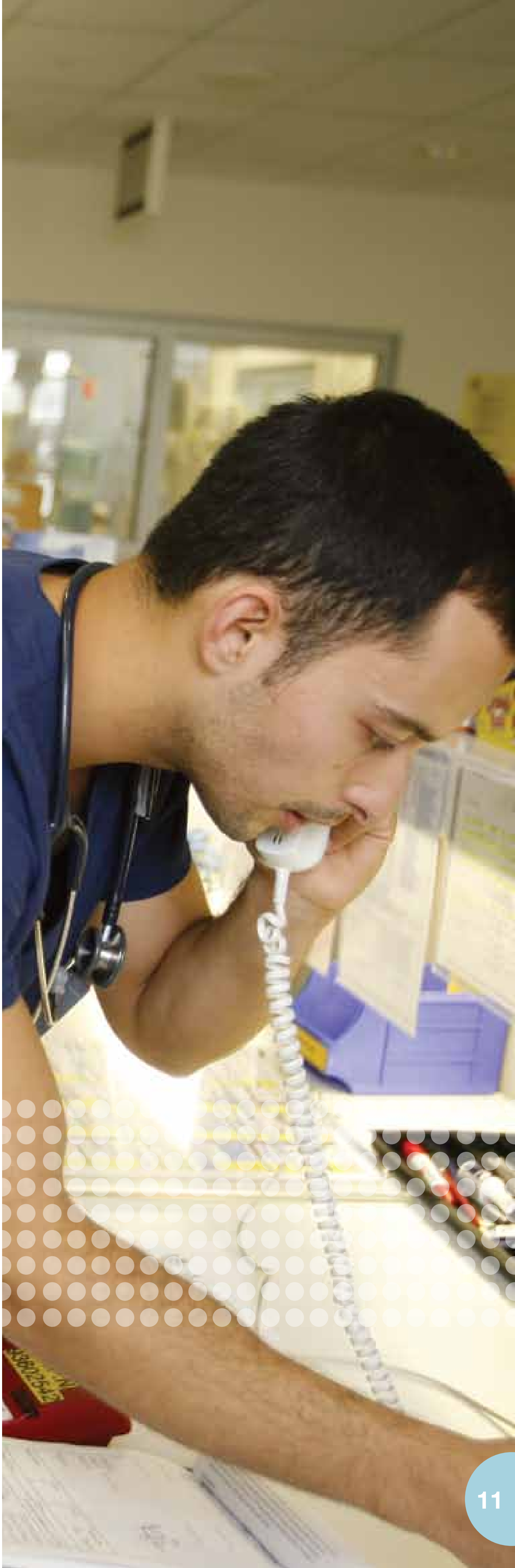

3.9 All opportunities should be taken by the clinicians providing emergency assistance to use the call as an educational opportunity for ward staff and students.

3.10 The clinicians providing emergency assistance should communicate in an appropriate, detailed and structured way with the attending medical officer or team about the consequences of the call, including documenting information in the health care record.

3.11 Events surrounding the call for emergency assistance and actions resulting from the call should be documented in the health care record and considered as part of ongoing quality improvement processes.

4 Clinical communication

Effective communication and team work among clinicians is an essential requirement for recognising and responding to clinical deterioration. Poor communication at handover and in other situations has been identified as a contributing factor to incidents where clinical deterioration is not identified or properly managed. A number of structured communication protocols exist that can be used for handover and as part of ongoing patient management.

4.1 Formal communication protocols should be used to improve the functioning of teams when caring for a patient whose condition is deteriorating.

4.2 The value of information about possible deterioration from the patient, family or carer should be recognised.

4.3 Information about deterioration should be communicated to the patient, family or carer in a timely and ongoing way.

4.4 There should be adequate communication and discussion about the wishes of the patient regarding advance care planning, resuscitation and other active treatment.

4.5 Structured handover processes, including documentation of handovers, should be used for all patients.

4.6 The handover protocol used should include information about the most recent observations and clinical assessment.

4.7 Handover procedures should include the identification of patients who are deteriorating and communication of information that is relevant to their management.

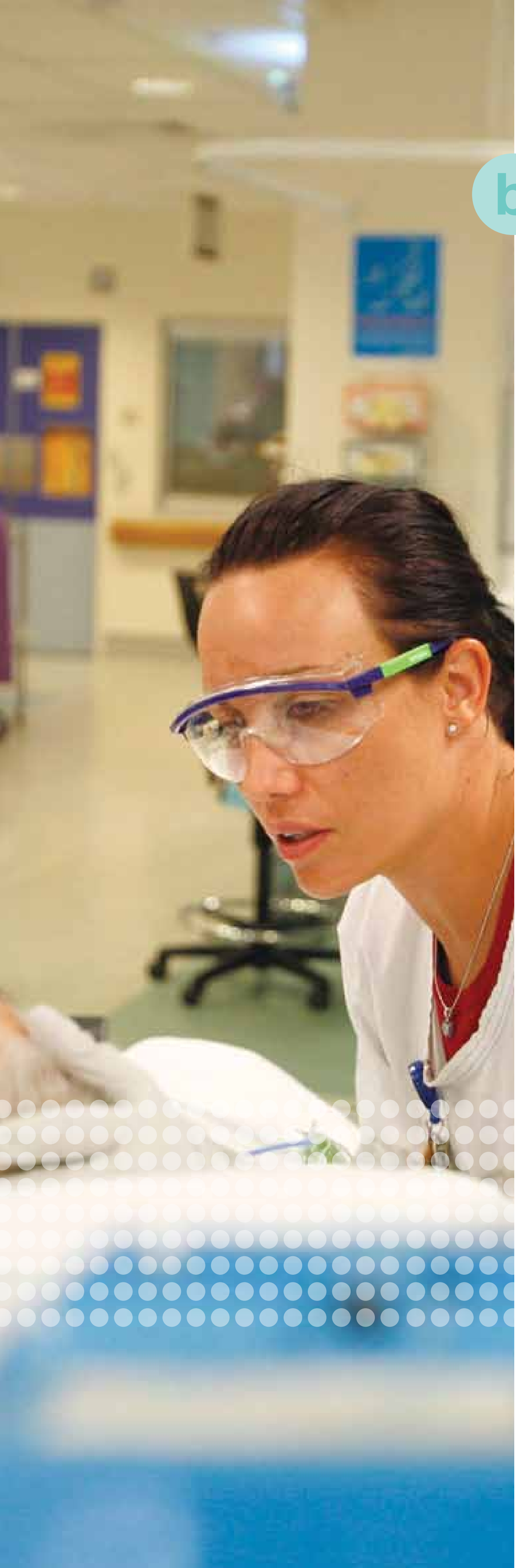

b organisational prerequisites

5 Organisational supports

Recognition and response systems should be part of standard clinical practice. Nonetheless, the introduction of new systems to optimise care of patients whose condition is deteriorating requires organisational support and executive and clinical leadership for success and sustainability.

5.1 A formal policy framework regarding recognition and response systems should exist and should include issues such as:

- governance arrangements
- roles and responsibilities
- communication processes
- resources for the rapid response system, such as staff and equipment
- training requirements
- evaluation, audit and feedback processes
- arrangements with external organisations that may be part of the rapid response system

5.2 This policy framework should apply across the acute health care facility, and identify the planned variations in the escalation protocol and responses that might exist in different circumstances (such as for different times of day).

5.3 Any new recognition and response systems or procedures should be integrated into existing organisational and safety and quality systems to support their sustainability and opportunities for organisational learning.

5.4 Recognition and response systems should encourage staff to react positively to escalation of care, irrespective of circumstances or outcome.

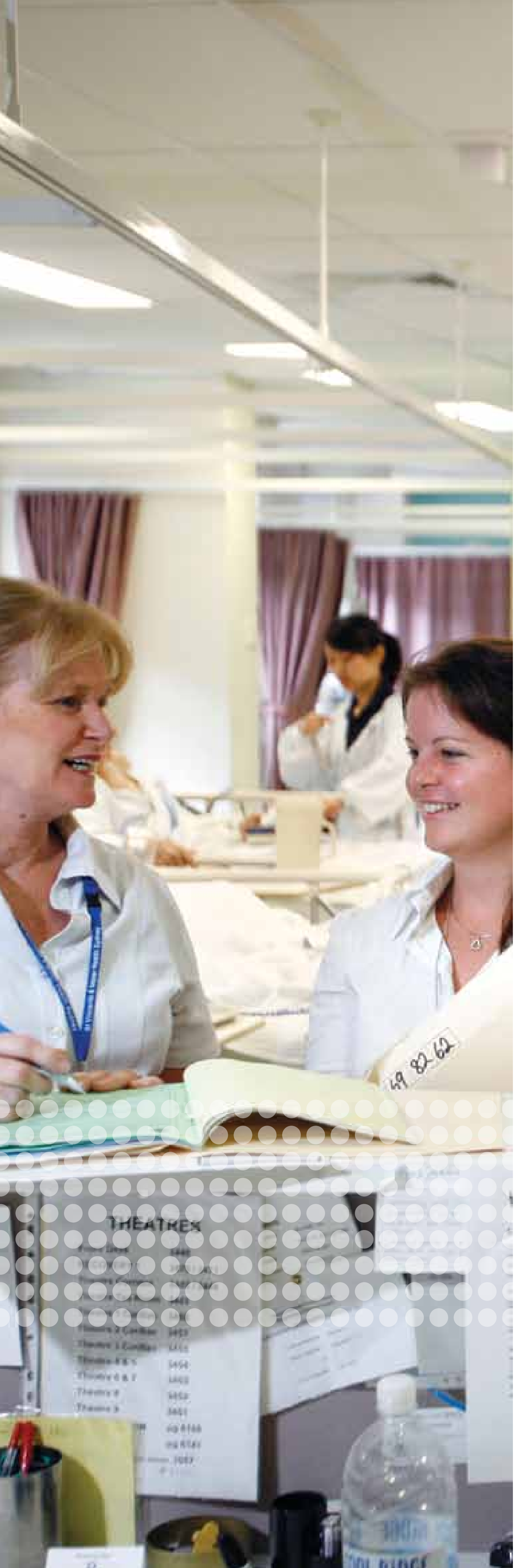

5.5 Appropriate policies and documentation regarding advance care directives, treatment-limiting decisions and end-of-life decision making are critical in ensuring that the care delivered in response to deterioration is consistent with appropriate clinical practice and the patient's expressed wishes.

5.6 A formal governance process (such as a committee) should oversee the development, implementation and ongoing review of recognition and response systems. If a committee has this role, it should:

- have appropriate responsibilities delegated to it, and be accountable for its decisions and actions
- monitor the effectiveness of interventions and education
- have a role in reviewing performance data
- provide advice about the allocation of resources
- include consumers, clinicians, managers and executives

5.7 Organisations should have systems in place to ensure that the resources required to provide emergency assistance (such as equipment and pharmaceuticals) are always operational and available.

6 Education

Having an educated and suitability skilled and qualified workforce is essential to provide appropriate care to patients whose condition is deteriorating. Education should cover knowledge of observations and identification of clinical deterioration, as well as appropriate clinical management skills. Skills such as communication and effective team work are needed to provide appropriate care to patients whose condition is deteriorating, and should also be part of staff development. The education programs provided by an individual facility should be consistent with the needs

and resources of the organisation, and could be standardised within areas, regions or jurisdictions.

6.1 All clinical and non-clinical staff should receive education about the local escalation protocol relevant to their position. They should know how to call for emergency assistance if they have any concerns about a patient, and know that they *should* call under these circumstances. This information should be provided at the commencement of employment and as part of regular refresher training.

6.2 All doctors and nurses should be able to:

- systematically assess a patient
- understand and interpret abnormal physiological parameters and other abnormal observations
- initiate appropriate early interventions for patients who are deteriorating
- respond with life-sustaining measures in the event of severe or rapid deterioration, pending the arrival of emergency assistance
- communicate information about clinical deterioration in a structured and effective way to the attending medical officer or team, to clinicians providing emergency assistance and to patients, families and carers
- understand the importance of, and discuss, end-of-life care planning with the patient, family and/or carer
- undertake tasks required to properly care for patients who are deteriorating, such as developing a clinical management plan, writing plans and actions in the health care record and organising appropriate follow up

6.3 As part of the rapid response system, competency in advanced life support should be ensured for sufficient clinicians who provide emergency assistance to guarantee access to these skills according to local protocols.

6.4 A range of methods should be used to provide the required knowledge and skills to staff. These may include provision of information at orientation and regular refreshers using face-to-face and online techniques, as well as simulation centre and scenario based training.

7 Evaluation, audit & feedback

Evaluation of new systems is important to establish their efficacy and determine what changes might be needed to optimise performance. Ongoing monitoring is necessary to track changes in outcomes over time and to check that these systems are operating as planned.

7.1 Data should be collected and reviewed locally and over time regarding the implementation and effectiveness of recognition and response systems.

7.2 Recognition and response systems should be evaluated to determine whether they are operating as planned. Evaluation may include checking the existence of required documentation, policies and protocols (such as the escalation protocol) and compliance with policy (such as completion rates of observation charts or proportion of staff who have received mandatory training).

7.3 Systems should be evaluated to determine whether they are improving the recognition of and response to clinical deterioration. Evaluation may include collecting and reviewing data about calls for emergency assistance, and adverse events such as cardiac arrests, unplanned admissions to intensive care and unexpected deaths.

7.4 The following data should be collected for each call for emergency assistance that is made to the rapid response system:

- patient demographics
- date and time of call, response time and stand down time
- the reason for the call
- the treatment or intervention provided
- outcomes of the call, including disposition of the patient

This information, as well as information about reviews conducted by the attending medical officer or team, should be included in the health care record.

7.5 Regular audits of triggers and outcomes should be conducted for patients who are the subject of calls for emergency assistance. Where these data are available, this could include longer-term outcomes for patients (such as 30 and 60 day mortality).

7.6 Evaluation of the costs and potential savings associated with recognition and response systems could also be considered.

7.7 Information about the effectiveness of the recognition and response systems may also come from other clinical information such as incident reports, root cause analyses, cardiac arrest calls and death reviews. A core question for every death review should be whether the escalation criteria for the rapid response system was met, and whether care was escalated appropriately.

7.8 As part of the implementation of new systems, feedback should be obtained from frontline staff about the barriers and enablers to change. Issues and difficulties regarding implementation should be considered for different settings.

7.9 Consistent with any implementation process, information collected as part of ongoing evaluation and audit should be:

- fed back to ward staff and the attending medical officer or team regarding their own calls for emergency assistance
- fed back to the clinicians providing emergency assistance
- reviewed to identify lessons that can improve clinical and organisational systems
- used in education and training programs
- used to track outcomes and changes in performance over time

7.10 Indicators of the implementation and effectiveness of recognition and response systems should be monitored at senior governance levels within the organisation (such as by senior executives or relevant quality committees).

8 Technological systems and solutions

In health care, new technologies are constantly being developed that have the potential to improve the safety and quality of care. Some of these are relevant to recognition and response systems, including the use of hand-held computers to collect observations, automatic monitoring of observations and automatic alerts where clinical trigger points are reached. Technology needs to be introduced in such a way that it supports the work of clinicians providing care to patients. The potential risks of technological systems also need to be understood and managed.

8.1 Recognition and response systems should consider the inclusion of technological solutions based on evidence of efficacy and cost, as well as consideration of possible additional safety and quality risks. Unintended adverse effects should be considered by explicit study during implementation.

8.2 Technological solutions should not place a barrier between the clinician and the patient; instead they should enhance the care process and interaction.

8.3 Where technological solutions are introduced, the recognition and response systems should still conform to the elements specified in this Consensus Statement.

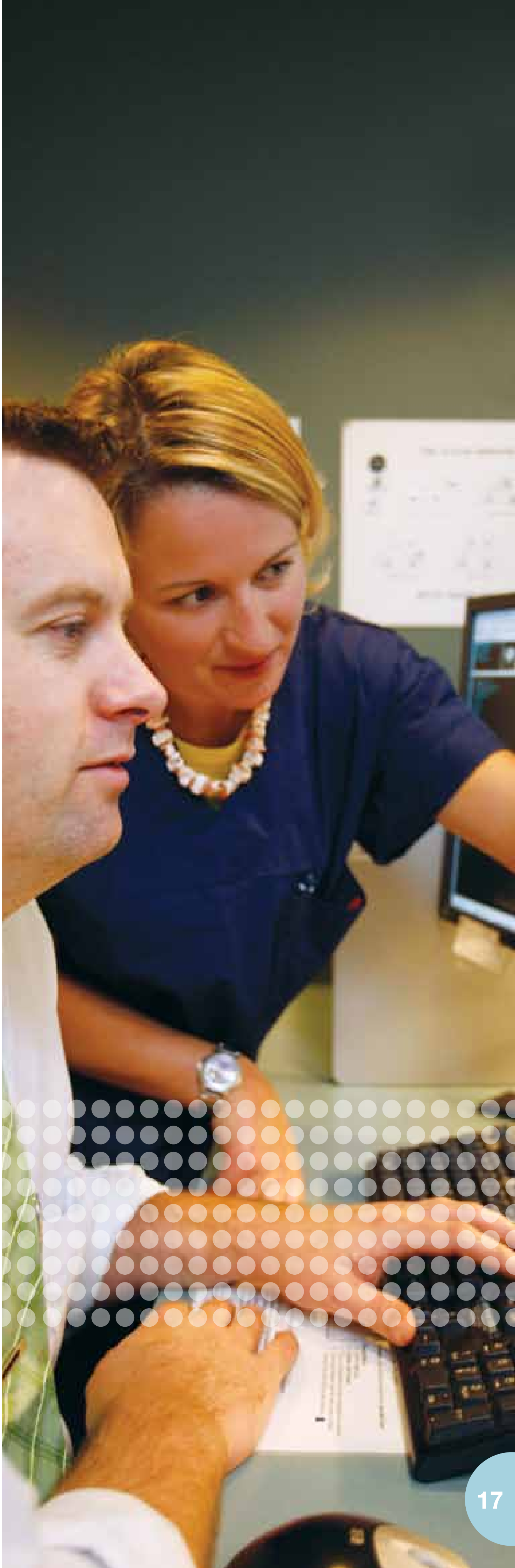

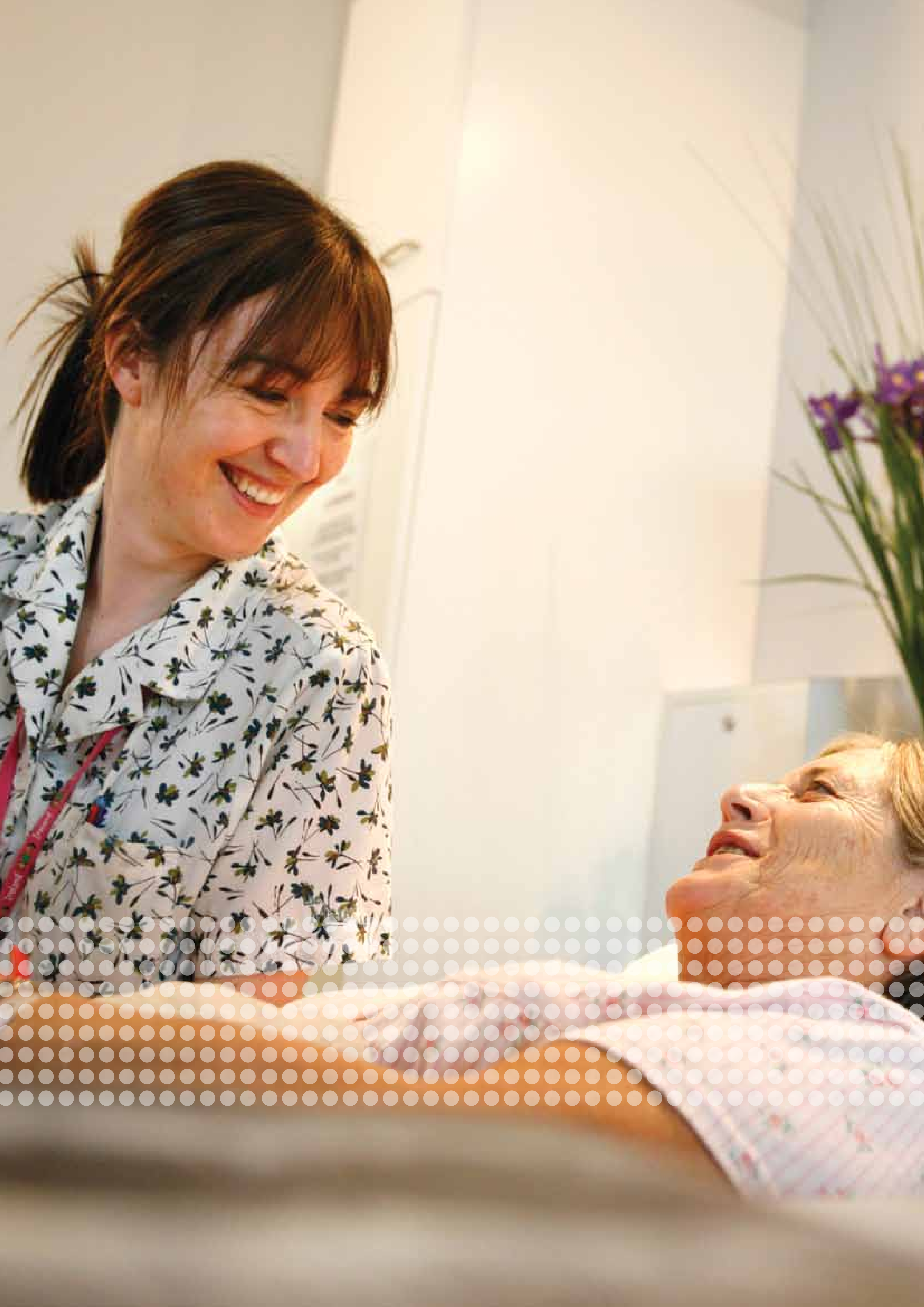

glossary

Acute health care facility: A hospital or other health care facility providing health care services to patients for short periods of acute illness, injury or recovery.

Advance care directive: Instructions that consent to, or refuse the future use of specified medical treatments (also known as a health care directive, advance plan or other similar term).

Advanced life support: The preservation or restoration of life by the establishment and/or maintenance of airway, breathing and circulation using invasive techniques such as defibrillation, advanced airway management, intravenous access and drug therapy.

Attending medical officer or team: The treating doctor or team with primary responsibility for caring for the patient.

Definitive disposition: The location, such as a ward or hospital, to which the patient will be transferred after initial stabilisation.

Definitive care: The clinical care required to maintain the stabilisation achieved and, where possible, to restore the patient to health.

Emergency assistance: Clinical advice or assistance provided when the patient's condition has deteriorated severely. This assistance is provided as part of the rapid response system, and is additional to the care provided by the attending medical officer or team.

Escalation protocol: The protocol that sets out the organisational response required for different levels of abnormal physiological measurements or other observed deterioration. The protocol applies to the care of all patients at all times.

Monitoring plan: A written plan that documents the type and frequency of observations to be recorded.

Rapid response system: The system for providing emergency assistance to patients whose condition is deteriorating. The system will include the clinical team or individual providing emergency assistance, and may include on-site and off-site personnel.

Recognition and response systems: Formal systems to support staff to promptly and reliably recognise patients who are clinically deteriorating, and to respond appropriately to stabilise the patient.

Treatment-limiting decisions: Decisions that involve the reduction, withdrawal or withholding of life-sustaining treatment. These may include 'no cardiopulmonary resuscitation' (CPR), 'not for resuscitation' and 'do not resuscitate' orders.

contributing documents

Clinical Excellence Commission, Greater Metropolitan Clinical Taskforce, NSW Department of Health. *Between the Flags Project: The Way Forward* 2008, http://www.cec.health.nsw.gov.au/resources/betweentheflags/TheWayForward.pdf, accessed 19 December 2008.

Department of Health. *Competencies for Recognising and Responding to Acutely Ill Patients in Hospital* 2009, http://www.dh.gov.uk/en/Consultations/Liveconsultations/DH_083630, accessed 3 April 2009.

Department of Human Services. *Safer Systems – Saving Lives, Implementing a Rapid Response System* 2006, http://www.health.vic.gov.au/sssl/downloads/rrs_toolkit.pdf, accessed 15 April 2008.

DeVita MA, Bellomo R, Hillman K, et al. Findings of the First Consensus Conference on Medical Emergency Teams. *Critical Care Medicine*. 2006; 34(9): pp 2463–78.

Institute for Health Improvement, *Getting Started Kit: Rapid Response Teams* 2008, http://www.ihi.org/IHI/Programs/Campaign/RapidResponseTeams.htm, accessed 27 January 2009.

Jacques T, Fisher M, Hillman K, Berry M, Hughes C, Lam D, Manasiev B, Morris R, Nguyen N, Pandit R, Pile A, Saul P. DETECT Manual: Detecting deterioration, evaluation, treatment, escalation and communicating in teams. Clinical Excellence Commission & NSW Health, 2009.

Joint Commission on Accreditation of Healthcare Organisations, *National Patient Safety Goals* 2008, http://www.jointcommission.org/NR/rdonlyres/31666E86-E7F4-423E-9BE8-F05BD1CB0AA8/0/HAP_NPSG.pdf, accessed 1 April 2009.

National Institute for Health and Clinical Excellence. *Acutely Ill Patients in Hospital: Recognition of and Response to Acute Illness in Adults in Hospital* 2007, http://www.nice.org.uk/nicemedia/pdf/CG50FullGuidance.pdf, accessed 24 January 2008.

National Patient Safety Agency, *Recognising and Responding Appropriately to Early Signs of Deterioration in Hospitalised Patients* 2007, http://www.npsa.nhs.uk/nrls/alerts-and-directives/directives-guidance/acutely-ill-patient/deterioration-in-hospitalised-patients/ accessed 11 March 2008.

NHS Patient Safety First Campaign, *The 'How to Guide' for Reducing Harm From Deterioration* 2008, http://www.patientsafetyfirst.nhs.uk/ashx/Asset.ashx?path=/How-to-guides-2008-09-19/Deterioration%201.1_17Sept08.pdf, accessed 1 April 2009.

NSW Department of Health. Recognition and Management of a Patient who is Clinically Deteriorating (PD2010_026). NSW Department of Health: Sydney, 2010, http://www.health.nsw.gov.au/policies/pd/2010/pdf/PD2010_026.pdf, accessed 14 May, 2010.

Peberdy MA, Cretikos M, Abella BS, et al. Recommended guidelines for monitoring, reporting and conducting research on medical emergency team, outreach and rapid response systems: An Utstein-style scientific statement. *Circulation*. 2007; 116: pp 2481–2500.

Sebat F (Ed.) *Designing, Implementing and Enhancing a Rapid Response System*. Society of Critical Care Medicine, January 2009.

Veteran's Health Administration, *Rapid Response Teams: A Bridge Over Troubled Waters* 2007, https://www.vha.com/Solutions/ClinicalImprovement/Documents/rrt_final.pdf, accessed 3 April 2009.

Australian Commission on
Safety and Quality in Health Care
Level 7, 1 Oxford Street
Darlinghurst NSW 2010
telephone 02 9263 3633
email mail@safetyandquality.gov.au
